# Extensive wet episodes in Late Glacial Australia resulting from high-latitude forcings

**DOI:** 10.1038/srep44054

**Published:** 2017-03-08

**Authors:** Germain Bayon, Patrick De Deckker, John W. Magee, Yoan Germain, Sylvain Bermell, Kazuyo Tachikawa, Marc D. Norman

**Affiliations:** 1 IFREMER, Unité de Recherche Géosciences Marines, Brest, France; 2 Royal Museum for Central Africa, Department of Earth Sciences, Tervuren, Belgium; 3 The Australian National University, Research School of Earth Sciences, Canberra, Australia; 4 Aix Marseille University, CNRS, IRD, Collège de France, CEREGE, Aix-en-Provence, France

## Abstract

Millennial-scale cooling events termed Heinrich Stadials punctuated Northern Hemisphere climate during the last glacial period. Latitudinal shifts of the intertropical convergence zone (ITCZ) are thought to have rapidly propagated these abrupt climatic signals southward, influencing the evolution of Southern Hemisphere climates and contributing to major reorganisation of the global ocean-atmosphere system. Here, we use neodymium isotopes from a marine sediment core to reconstruct the hydroclimatic evolution of subtropical Australia between 90 to 20 thousand years ago. We find a strong correlation between our sediment provenance proxy data and records for western Pacific tropical precipitations and Australian palaeolakes, which indicates that Northern Hemisphere cooling phases were accompanied by pronounced excursions of the ITCZ and associated rainfall as far south as about 32°S. Comparatively, however, each of these humid periods lasted substantially longer than the mean duration of Heinrich Stadials, overlapping with subsequent warming phases of the southern high-latitudes recorded in Antarctic ice cores. In addition to ITCZ-driven hydroclimate forcing, we infer that changes in Southern Ocean climate also played an important role in regulating late glacial atmospheric patterns of the Southern Hemisphere subtropical regions.

During the last glacial, episodes of massive iceberg discharges in the Northern Hemisphere were accompanied by substantial cooling and weakening of the Atlantic Meridional Oceanic Circulation (AMOC)^1^, leading ultimately to out-of-phase warming periods in Antarctica^2^,^3^. These abrupt climate change events, known as Heinrich Stadials (HS), were also rapidly transferred southward via latitudinal migrations of the ITCZ, as directly inferred from proxy records indicating reduced precipitation in northern tropics and subtropical regions^4^,^5^, and increased monsoon rainfall in the Southern Hemisphere^6^,^7^. This interhemispheric atmospheric seesaw is thought to have caused global displacement of air masses, with presumably major impact on higher-latitude climates in the Southern Hemisphere^8^,^9^ and global ocean circulation patterns^2^,^10^,^11^. To date however, it is unclear whether the evolution of Southern Hemisphere climates during the last glacial period was mainly driven by northern (via ITCZ latitudinal variations) and/or southern high-latitude climate forcing.

To provide new insights on this issue, we have analysed a well-dated^12^ marine sediment core (MD03-2607; 36°57.64′S, 137°24.39′E; 865 m water depth) recovered from near the mouth of the River Murray, the end point of the Murray-Darling Basin (MDB), Australia’s largest river system (Fig. 1). The MDB lies in the subtropical climate zone between about 25°S and 37°S, displaying a marked latitudinal gradient of contrasting geological and climatic settings. In the northern part of the basin (~25–32°S), an area with weak dominance of ITCZ-driven summer monsoon rainfall, the Darling River sub-basin drains large outcrops of Mesozoic and Cenozoic terrains. In contrast, the southern River Murray sub-basin (~32–37°S) is dominated by Palaeozoic rocks, and is more strongly influenced by winter precipitation associated with Southern Hemisphere westerly winds (SHWW). As a consequence, the use of geochemical proxies allowing discrimination between Darling *versus* Murray sources in sedimentary records of ancient MDB discharge can provide continuous and integrated information on past hydroclimate variability in subtropical Australia, and its potential link to ITCZ and SHWW strength and/or latitudinal shifts.

We reconstructed the composition of past sediment discharge using neodymium isotopic ratios (^143^Nd/^144^Nd, or ε_Nd_ in epsilon notation), focusing on the fine-grained (<2 μm) clay-rich detrital fraction of the sediment. In addition to being exported from river basins with presumably minimum transfer time, clay-size fractions are also less prone than coarser sedimentary particles to mineralogical sorting and hence better suited for provenance studies, especially near upwind arid source areas. The three main potential contributors of fine-grained sediments to the studied site, i.e. the Darling River, the River Murray and southern Australian dust-source regions, are characterized by distinctive Nd isotopic signatures^13^,^14^ (ε_Nd_ = −2.4 ± 2.4, 1 SD; −9.5 ± 0.9 and −12.0 ± 2.9, respectively; Fig. 2; Table S1), which makes Nd isotopes particularly well-suited for provenance studies in this area (Fig. 1). Neodymium isotopes remain largely unaffected by erosion and transport processes^15^, so that the average ε_Nd_ composition of each potential provenance area is expected to have remained relatively unchanged over the last glacial period.

The ε_Nd_ detrital record of core MD03-2607 indicates highly variable contributions from MDB sources and southern Australian dusts, starting, from about 86 to 68 thousand years before present (kyr BP), with a pronounced shift towards more radiogenic Nd isotopic compositions (from about ε_Nd_ = −10 to −6; Fig. 2). This trend reflects enhanced contribution from Darling river-borne material and reduced inputs from southern Australia dusts and/or River Murray particles. During that period, sea-level dropped by about 50 meters (Fig. 2), resulting in a progressive ‘migration’ of the proximity of the studied area to the Murray mouth, and producing a more efficient sediment transfer from the MDB to site MD03-2607 (ref. 16). An inverse trend from ε_Nd_ ~−5 to −13 also characterizes the last deglaciation period at the nearby site MD03-2611 (Fig. 2), coinciding with an almost 90-m steep sea-level rise between ~17 and 9 kyr BP and also interpreted in terms of overwhelming contribution of aeolian dust particles relative to riverine detrital inputs from the MDB (ref. 16). Overall, the observed ε_Nd_ trend between about 90 and 20 ka correlates well with palaeoprecipitation records for the tropical western Pacific region^5^,^17^,^18^,^19^ (Fig. 3a,b,c). Rainfall patterns in the tropics are strongly controlled by the position and intensity of the ITCZ, which varies on seasonal timescales from north to south of the Equator (Fig. 1). During the last ice age, large southward ITCZ migrations have accompanied both the onset of MIS-4 and the abrupt millennial-scale HS events^20^. In the northwest Pacific region, these ITCZ shifts are illustrated by abrupt drops in precipitation recorded both by Borneo speleothems^5^ (Fig. 1; Fig. 3a) and, farther south (~3°S), at the northern Papua New Guinea (PNG) margin (site MD05-2920; Fig. 1), as inferred from substantial decreases of sedimentary Ti/K ratios during HS-3-6-7b events^17^,^18^ (Fig. 3b). At site MD05-2920, Ti/K ratios have been interpreted as a proxy for the presence of coarse-grained river-borne terrigenous material, presumably increasing during past wet periods when intensifying rainfall caused more intense erosion and export of coarser-grained riverine particles to the ocean, and vice versa^18^. For the same time intervals, foraminifera trace element proxy data suggest episodes of enhanced river discharge in southern PNG (~9°S; Fig. 1). The use of Nd/Ca ratios in planktonic foraminifera at this latter southern PNG site (MD05-2925; Fig. 3c), taken as an indicator for the intensity of REE-rich dissolved riverine inputs from Papua New Guinean rivers, shows that the sudden drops in precipitation recorded at Borneo and northern PNG were accompanied by more intense rainfall in the southerly tropical regions^19^. This pattern agrees well with results obtained from dust flux reconstructions in the central equatorial Pacific^21^, which provide evidence for a major latitudinal shift of the ITCZ by at least 4 degrees in this area during an earlier HS event. Within the uncertainties of dating (see Supplementary Information), our ε_Nd_ provenance profile for palaeo-MDB sediments matches remarkably well with the southern PNG river-discharge record. In core MD03-2607, the periods of HS events and glacial maxima (Marine Isotope Stage 4 and MIS-2) correspond to more radiogenic ε_Nd_ signatures (Fig. 3d), indicating enhanced sediment input from the Darling River watershed. While a reduction in SHWW in the River Murray sub-basin could possibly account for the observed trend, the good agreement between MDB and southern PNG proxy records seems more consistent with southward ITCZ migrations leading to intensifying rainfall in the northern Darling River sub-basin relative to the southern MDB area.

Our proxy data are also consistent with palaeoclimatic records for Australian lakes^22^,^23^,^24^ (Fig. 3e). The palaeoshoreline reconstruction of Lake Eyre, the largest central Australia inland basin draining summer-monsoon-fed watersheds (Fig. 1), indicate a trend towards generally dryer conditions since the last interglacial (MIS-5) period, punctuated with alternating periods of high and low (or dry) lake levels^22^. Lake Frome, a separate depocentre of the same basin, also displays some high lake shorelines, though not always synchronous with Lake Eyre levels^23^. Despite the paucity of dates and the poor constraints on the timing of upper and lower boundaries of wet/dry lake phases (inherent to low-precision luminescence-derived chronologies), the presumed periods of high lake stands are consistent with inferred episodes of southward ITCZ migrations, as far as about 32°S south (i.e. the boundary between the two Darling and Murray sub-basins), and associated shifts towards more radiogenic ε_Nd_ signatures recorded at site MD03-2607.

In contrast to the abrupt seesaw-like tropical precipitation records of Borneo during HS events, the ε_Nd_ profile for MDB sediment provenance suggests instead a more gradual evolution of rainfall in subtropical Australia. In particular, the ε_Nd_ shifts towards more radiogenic values associated with every major southward excursions of the ITCZ appear to have started earlier, and persisted later, than the corresponding HS events as recorded in Borneo (Fig. 4). There are clear inherent uncertainties when establishing the chronostratigraphy of marine sediment cores beyond the limit of radiocarbon (~50 kyr BP), which prevent any detailed comparison with more precise U-Th-dated speleothem records. In addition, possible temporal biases in our detrital ε_Nd_ record may exist due to temporary sediment storage within the drainage basin and/or post-depositional bioturbation effect. Nevertheless, bearing in mind these potential limitations, the relatively good agreement observed between our ε_Nd_ profile and PNG river-discharge/Australian lake records still suggests that wet episodes in late glacial Australia lasted substantially longer (between ~3 to >5 ka each, as visually estimated from both MD03-2607 and palaeolake data^22^,^23^,^24^) than the average duration of the southward ITCZ migrations associated with HS events (<2 ka; as inferred from Borneo^5^ and Chinese^6^ cave monsoon records; Fig. 4). All the above suggests therefore that rainfall patterns in subtropical Australia during the last glacial period were modulated by additional mechanisms, rather than being simply driven by ITCZ-driven Northern Hemisphere forcing.

In fact, our ε_Nd_ proxy record for hydroclimate variability in Australia also exhibits strong similarities with the late glacial evolution of Antarctic temperatures^25^ (Fig. 4d), hence suggesting a possible link to southern high-latitude forcing. Except for MIS-4, the most radiogenic ε_Nd_ values observed between 35 and 65 kyr BP (i.e. indicative of higher sediment contributions from the Darling River and tributaries relative to the River Murray sub-basin) appear to coincide with the warm phases recorded in Antarctic ice-cores that followed the onset of HS events (Fig. 4d). Antarctic climate variability over the last ice age is thought to have been closely connected to Northern Hemisphere forcing too, with gradual warming periods being generally attributed to enhanced heat storage in the Southern Hemisphere, in response to AMOC collapse during HS events and other abrupt cold stadials^3^. During these periods, Antarctic warming may have also been promoted through atmospheric teleconnections, via ITCZ-driven southward shifts of the SHWW belt^2^,^10^. In turn, the evolution of Antarctic climate during the last ice age most probably played a major role in controlling the position of the Subtropical Front in the Southern Ocean^26^, with presumably direct impact on the position/intensity of SHWW and associated rainfall. This complex interhemispheric interplay is illustrated by a δ^18^O stalagmite record from New Zealand’s South Island (~42°S)^9^, which reveals that wet periods in the Southern Hemisphere mid-latitudes prevailed at times of southward ITCZ migrations, but also when cooler conditions in Antarctica led to northward shifts of the STF and strengthening of the SHWW (Fig. 4c). Previous studies have already argued for a connection between high-latitude climate changes in the Southern Hemisphere and the hydrologic evolution of monsoon-dominated regions during the Late Quaternary period^7^,^27^,^28^,^29^. Using high-resolution elemental profiles in a series of sediment cores from the Timor Sea, Kuhn *et al*. (ref. 27) proposed that the onset of Antarctic warming that followed the HS1 climate event led to a southward shift of the ITCZ over northwestern Australia. A detailed comparison of the climate variability recorded in the Hulu Cave and polar ice-cores also led Rohling *et al*. (ref. 28) to suggest that millennial-scale monsoon variability was dominated by Southern Hemisphere climate change during glacial times, when monsoon was weak overall. Taken together, the above-mentioned studies provide support that the late glacial hydroclimate variability of subtropical Australia, as inferred from our ε_Nd_ profile for core MD03-2607, was probably driven by combined influences of both northern- (via ITCZ) and southern (via SHWW) high-latitude forcings. For instance, it is possible that the poleward shifts of the Subtropical Front during warm periods in Antarctica, which led to temporary southward atmospheric displacement of the SHWW belt, also acted as a factor delaying the contemporaneous northward retreat of the ITCZ. Alternatively, enhanced greenhouse forcing associated with the episodic rises of atmospheric CO_2_ that accompanied the Antarctic warming phases could have resulted in enhanced heat low-pressure cells over Australia, thereby accentuating the southward ‘pull’ of the ITCZ across the SH mid-latitudes^27^. While the exact mechanism remains elusive, these interactions between northern and southern high-latitude climates most likely drove important fluctuations of interhemispheric temperature/pressure gradients^28^, which may have resulted in protracted rainfall episodes in the Southern Hemisphere subtropical regions during the last glacial period.

## Methods

### Chemical preparation and Nd isotope analyses

The detailed description of chemical preparation and analytical methods can be found elsewhere^15^. Briefly, about 3 g of bulk sediment were treated successively with 10% (v/v) acetic acid (AA), mixed 15% (v/v) AA and 0.05 M hydroxylamine hydrochloride (HH), and 5% hydrogen peroxide (H_2_O_2_) solutions, in order to remove any carbonate, Fe-Mn oxyhydroxide and organic components, respectively. The sequential leaching procedure used in this study was developed to minimize partial dissolution of the silicate component^30^. Recent work has shown however that easily alterable silicate minerals such as fresh volcanic components could undergo partial dissolution with the use of mixed AA-HH solutions^31^. In the case of our study, one cannot exclude that a minor proportion of the volcanogenic component hosted by Darling River-borne sediments dissolved during our second leaching step. Considering a simple mixing model between various proportions of Darling- versus Murray-borne sediments, and assuming arbitrarily that 10% of the Darling sediment endmember dissolved during the AA + HH leaching phase, the obtained Nd isotope composition of the resulting mixed sediment would be biased towards Murray endmember by less than 0.5 ε_Nd_ units. Importantly, this bias, while being proportional to the proportion of Darling River sediment, would not affect the observed downcore trend for Nd isotopes, and hence the conclusions of our study.

Clay-size fractions (about <2 μm) were then separated from the residual detritus by centrifugation. For Nd isotopic analyses, about 100 mg of clay-size sediment powder were digested by alkaline fusion. Neodymium isotopic measurements were performed at the Pôle Spectrométrie Océan (Brest, France) using a Thermo Scientific Neptune multi-collector ICPMS, after Nd purification by conventional ion chromatography. Mass bias corrections on Nd were made with the exponential law, using ^146^Nd/^144^Nd = 0.7219, and ^143^Nd/^144^Nd corrected values were normalized to a JNdi-1 value of ^143^Nd/^144^Nd = 0.512115. Repeated analyses of a JNdi-1 standard solution during the two measurement sessions of this study gave ^143^Nd/^144^Nd of 0.512106 ± 0.000005 (2 SD, n = 22) and 0.512121 ± 0.000009 (2 SD, n = 33), hence corresponding to an external reproducibility of ~ ± 0.10ε and ± 0.17ε (2 SD), respectively (Table S1). Epsilon Nd values (ε_Nd_) were calculated using ^143^Nd/^144^Nd = 0.512630 (ref. 32).

## Additional Information

**How to cite this article**: Bayon, G. *et al*. Extensive wet episodes in Late Glacial Australia resulting from high-latitude forcings. *Sci. Rep.* **7**, 44054; doi: 10.1038/srep44054 (2017).

**Publisher's note:** Springer Nature remains neutral with regard to jurisdictional claims in published maps and institutional affiliations.

## Supplementary Material

Supplementary Material

Supplementary Dataset 1

## Figures and Tables

**Figure 1:**
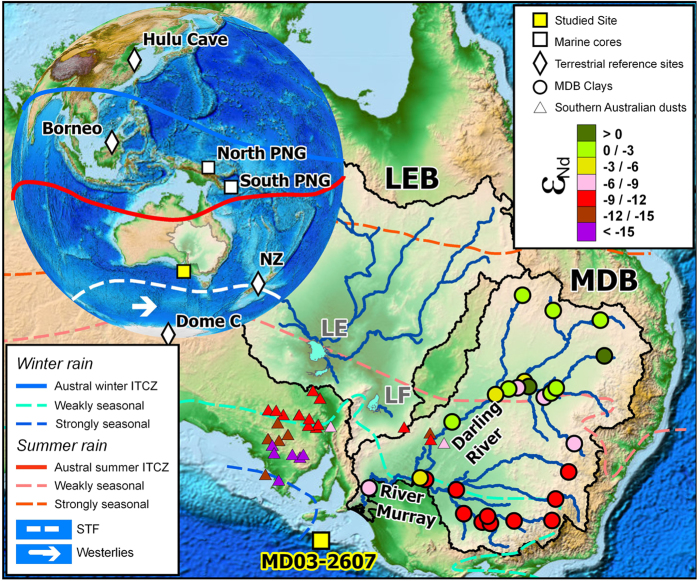
Study area and location of the studied core (MD03-2607). (**a**) Map of the western equatorial Pacific area, with position of the ITCZ in austral summer (Dec-Jan-Feb) and winter (June-July-Aug), inferred from NCEP/NCAR (http://www.cpc.ncep.noaa.gov/), and location of Hulu Cave^6^, Borneo^5^, New Zealand’s South Island^9^ speleothems, marine sediment^17^,^18^,^19^ and EPICA Dome C ice-core^25^ palaeoclimatic records used for comparison. The limits of influence for strongly and weakly summer-winter rainfall, as inferred from the seasonal precipitation ratio, are adapted from ref. 22. The version of Esri mapping software used is ArcGis Desktop advanced 10.3 (http://www.esrifrance.fr/arcgis.aspx). (**b**) Location of the Murray-Darling (MDB) and Lake Eyre (LEB) hydrological basins. The Darling River is a major tributary of the Murray-Darling system, with a total length of about 1500 km from its source to its confluence with the River Murray. The range of neodymium isotopic compositions (ε_Nd_) for MDB clay-size fractions^13^ (circles), and southern Australian dust sources^14^ (triangles) are also represented. LE and LF refer to Lake Eyre and Lake Frome, respectively.

**Figure 2:**
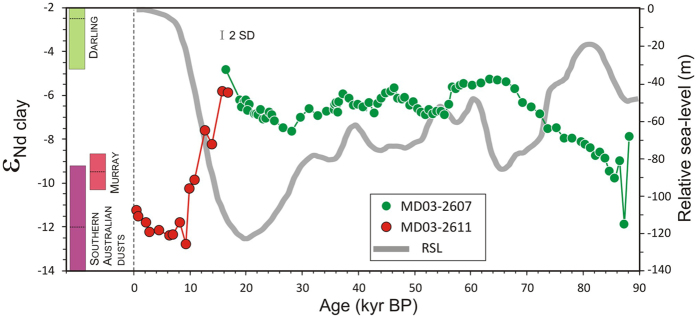
The last 90 ka evolution of neodymium isotopic ratios in clay-size detrital sediments at the SE Australian ocean margin. Neodymium isotope data (ε_Nd_) are reported for sites MD03-2607 (green circles; this study) and MD03-2611 (red circles; ref. 16), with the inferred uncertainty on measurements (±0.17 ε_Nd_; 2 SD). Also shown for comparison are the average ε_Nd_ compositions (±1 SD) for three main potential contributors of fine-grained sediments to the studied sites, i.e. the Darling River (−2.4 ± 2.4), the River Murray (−9.5 ± 0.9) and southern Australian dust-source regions (−12.0 ± 2.9). The relative sea-level curve (meters before present) for the last 90 kyr BP is also reported^33^.

**Figure 3:**
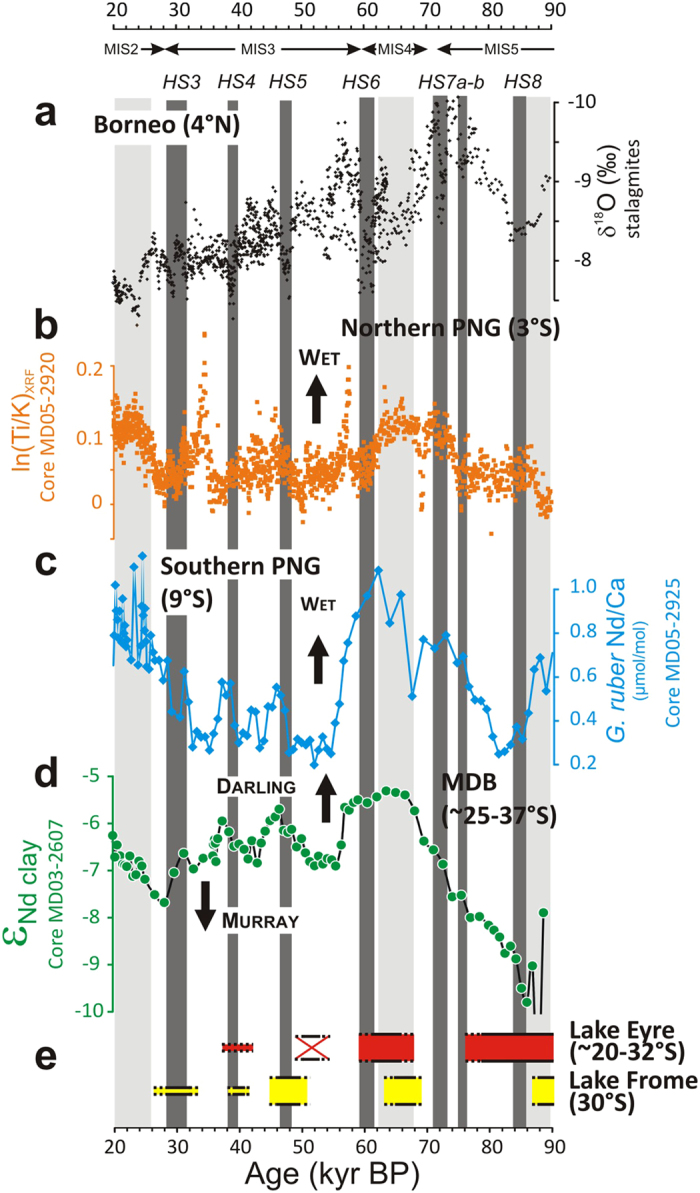
Proxy record for sediment provenance in MD03-2607 during the last glacial period, compared to other regional palaeoclimatic records. (**a**) δ^18^O record of western Pacific tropical precipitation from Borneo speleothems^5^. Note that abrupt shifts towards higher δ^18^O values (dry conditions) in Borneo stalagmites are attributed to southward ITCZ migrations. (**b**) XRF core scanner log-scale (Ti/K) intensity ratios for core MD05-2920, as a record of past sediment discharge at the northern Papua New Guinea (PNG) margin^17^. (**c**) Core MD05-2925 *G. ruber* Nd/Ca, as a record of past freshwater discharge at the southern PNG margin^19^. (**d**) Nd isotopes (ε_Nd_) in MD03-2607 representing relative sediment contributions from Darling River (−2.4 ± 2.4), River Murray (−9.5 ± 0.9), and southern Australian dust (−12.0 ± 2.9) source areas. **e**. Highstand records for major Australian lakes (Lake Eyre^22^, Lake Frome^23^), with bar thickness representing inferred lake-levels. Red-crossed bar indicates desiccation at Lake Eyre^22^ and dotted lines represent temporal uncertainties. Vertical dark and light grey bands indicate Heinrich Stadial (HS) events and the glacial maxima corresponding to Marine Isotope Stage (MIS) 2-4-5b, respectively.

**Figure 4:**
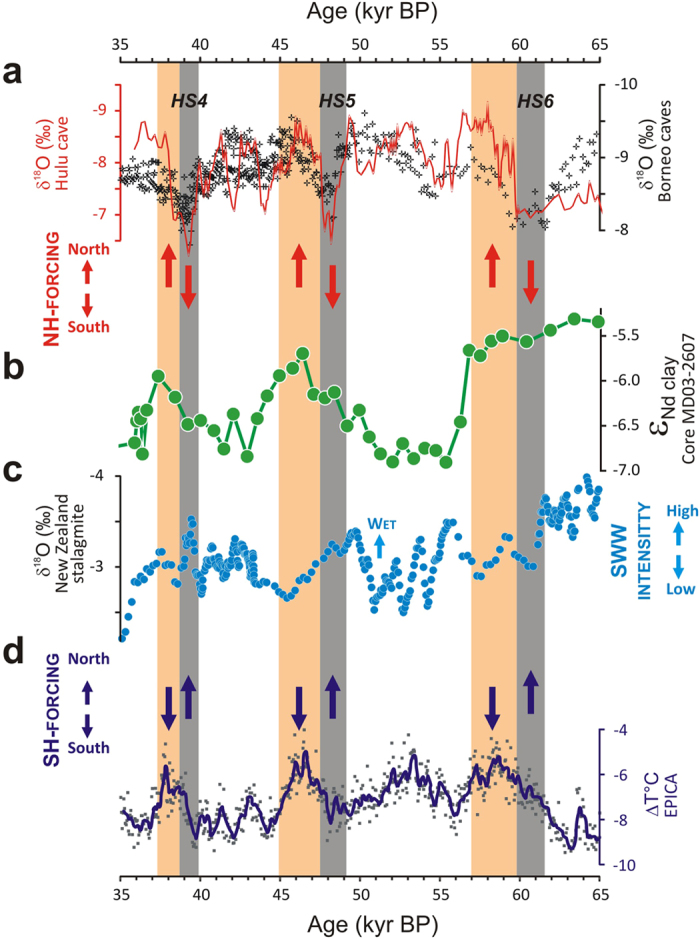
High-latitude forcings on hydroclimate patterns of the Southern Hemisphere subtropical regions between 35 and 65 kyr BP. (**a**) δ^18^O cave records for Chinese (Hulu Cave^6^) and Borneo speleothems^5^, in which abrupt δ^18^O drops indicate southward shifts of the ITCZ. (**b**) MD03-2607 ε_Nd_ proxy record for hydroclimate variability in subtropical Australia. (**c**) δ^18^O record for New Zealand’s South Island stalagmite^9^ (~42°S), representing the intensity of the Southern Hemisphere westerly wind (SHWW) belt. (**d**)Late glacial temperature evolution in Antarctica recorded at the EPICA Dome C ice-core^25^. ΔT°C represents the temperature difference from the average of the last 1000 years. The vertical arrows represent the presumed directions (north/south) of frontal shifts exerted by northern (red) and southern (blue) high-latitude forcings during the last glacial period. Vertical light grey and orange bands indicate Northern Hemisphere HS events and Antarctica Warm Intervals, respectively.
