# Supplementary material for: Extensive wet episodes in Late Glacial Australia resulting from high-latitude forcings
Source: Sci Rep. 2017 Mar 8;7:44054. doi: 10.1038/srep44054 (PMC5341032; doi:10.1038/srep44054)
Supplement: Supplementary Material [file srep44054-s1.pdf]

Supplementary information

Extensive wet episodes in Late Glacial Australia resulting from high-latitude forcings

Germain Bayon, Patrick De Deckker, John W. Magee, Yoan Germain, Sylvain Bermell, Kazuyo Tachikawa, Marc D. Norman

Comparison of oxygen isotope stratigraphy of the late glacial records of cores MD03-2607 (southeastern Australian margin), MD05-2925 (southern Papua New Guinea margin) and MD05-2920 (northern Papua New Guinea margin)

There are inherent uncertainties when establishing age models by tuning to the global benthic foraminifera $\delta^{18}O$ stack curve of Lisiecki and Raymo (2005). Cores MD05-2920 and MD05-2925 were recovered from similar water depths (1843m and 1661 m, respectively) and their benthic foraminifera $\delta^{18}O$ records display strong similarities. The age model for core MD05-2920, developed initially by Tachikawa et al. (2011) and Ménabréaz et al. (2012), has also been revised recently (Simon et al., 2016) using the Laschamp magnetic event (41,300 yr) as an independent age control point. For core MD03-2607 (the core analyzed during this study), the $\delta^{18}O$ stratigraphy was based on the *G. bulloides* planktonic foraminifera (Lopes dos Santos et al., 2013), but it also correlates relatively well with the LR04 stack curve of Lisiecki and Raymo (2005). Based on visual comparison, we roughly estimate an uncertainty of about 2 kyr between the three different sediment records. Within this uncertainty, we are confident that the relatively good agreement observed between the proxy records for the 3 cores is not the consequence of age model discrepancies, but instead most likely reflects the fact that they are mostly driven by similar climate forcings.

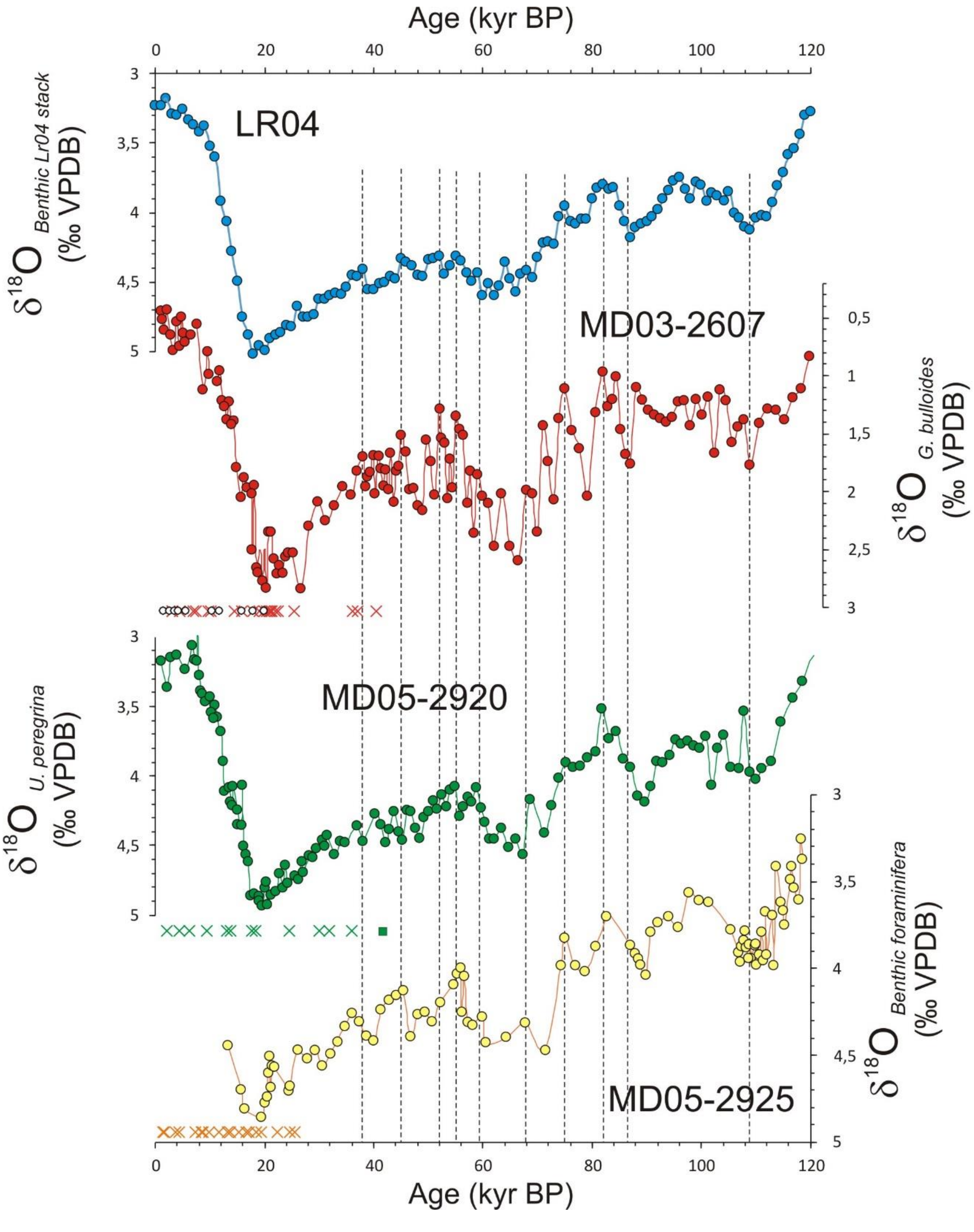

Figure S1: Foraminifera *$\delta^{18}O$ stratigraphy for the marine sediment records used in this study. The Lisiecki and Raymo's benthic $\delta^{18}O$ stack used for tuning is also shown for comparison. The dashed lines correspond to the age control points defined in core MD03-2607 by tuning with LR04 (Lopes dos Santos et al., 2012). The age models were also established using calibrated AMS C-14 dates (crosses; all cores), OSL dates (small white circles; core MD03-2607), and the Laschamp magnetic reversal (green square; core MD05-2920).*

Table S1. Neodymium isotopic composition of sediments from potential source regions in southeastern Australia

Sample	Lat	Long	$^{143}Nd/^{144}Nd$	ε_{Nd}		
**Darling River clay-size fractions (Gingele and De Deckker, 2005)**						
MDB2	-30,246	147,882	0,512504	**-2,5**		
MDB3	-30,017	148,121	0,512550	**-1,6**		
MDB6	-29,719	146,677	0,512454	**-3,4**		
MDB8	-29,948	146,864	0,512663	**0,6**		
MDB10	-30,057	145,951	0,512541	**-1,7**		
MDB11	-30,318	145,360	0,512350	**-5,5**		
MDB12	-31,560	143,378	0,512553	**-1,5**		
MDB15	-34,108	141,921	0,512429	**-3,9**		
MDB25	-25,793	146,586	0,512627	**-0,1**		
MDB26	-26,485	147,981	0,512544	**-1,7**		
MDB27	-26,927	150,132	0,512566	**-1,2**		
MDB28	-28,550	150,311	0,512689	**1,2**		
			**Average**	**-1,8**	**±**	**3,8**
**River Murray clay-size fractions (Gingele and De Deckker, 2005)**						
MDB14	-34,182	142,172	0,512087	**-10,6**		
MDB18	-34,567	139,595	0,512243	**-7,5**		
MDB19	-34,647	143,566	0,512159	**-9,2**		
MDB20	-35,735	143,910	0,512125	**-9,9**		
MDB21	-36,120	144,744	0,512119	**-10,0**		
MDB22	-36,176	145,119	0,512145	**-9,5**		
MDB23	-35,854	144,999	0,512119	**-10,0**		
MDB24	-36,067	146,202	0,512118	**-10,0**		
MDB29	-32,543	148,942	0,512215	**-8,1**		
MDB30	-33,834	148,684	0,512155	**-9,3**		
MDB31	-35,069	148,091	0,512133	**-9,7**		
MDB32	-36,046	147,931	0,512081	**-10,7**		
			**Average**	**-9,5**	**±**	**1,9**
**Southern Australian dust component exported to Murray Canyon Group area (i.e. clay-size fractions from core MD03-2611; Gingele et al., 2007)**						
MDB14			0,512038	**-11,5**		
MDB18			0,512023	**-11,8**		
MDB19			0,512010	**-12,1**		
MDB20			0,511987	**-12,5**		
			**Average**	**-12,0**	**±**	**0,4**

Epsilon Nd values (εNd) were recalculated using $^{143}Nd/^{144}Nd$ = 0.512630 (Bouvier et al., 2008).

Table S1. (Continued)

Sample	Lat	Long	$^{143}Nd/^{144}Nd$	ε_{Nd}		
**Southern Australian dusts (Ref. 14)**						
2,2	-31,760	137,848	0,512180	**-8,8**		
2.4	-31,798	137,186	0,512161	**-9,1**		
2,6	-31,668	137,277	0,512177	**-8,8**		
2,7	-31,666	137,276	0,512167	**-9,0**		
2,9	-31,707	136,828	0,512046	**-11,4**		
2,11	-31,399	136,870	0,512093	**-10,5**		
2,12	-30,471	137,357	0,512152	**-9,3**		
2.13a	-30,469	137,358	0,512164	**-9,1**		
2.13b	-30,469	137,358	0,512160	**-9,2**		
2,14	-30,124	137,036	0,512127	**-9,8**		
2.14a	-30,126	137,039	0,512212	**-8,2**		
2,15	-30,124	137,039	0,512157	**-9,2**		
2,17	-31,225	136,404	0,512012	**-12,1**		
2,18	-31,227	136,407	0,512061	**-11,1**		
2.19	-31,221	135,689	0,512071	**-10,9**		
2.20a	-31,218	135,691	0,512069	**-10,9**		
2.20b	-31,000	135,000	0,512081	**-10,7**		
2,21	-31,027	135,365	0,512069	**-10,9**		
2,24	-31,596	135,431	0,511990	**-12,5**		
2,25	-32,313	134,991	0,512018	**-11,9**		
2.26	-32,312	134,990	0,511896	**-14,3**		
2,27	-32,386	135,490	0,511955	**-13,2**		
2,28	-32,124	135,898	0,511941	**-13,4**		
2.30	-32,123	135,897	0,511956	**-13,1**		
2,32	-32,609	135,188	0,511750	**-17,2**		
2,34	-32,611	135,189	0,511742	**-17,3**		
2,35	-32,967	135,271	0,511767	**-16,8**		
2,36	-32,967	135,271	0,511785	**-16,5**		
2,38	-33,941	134,875	0,511942	**-13,4**		
2.40	-34,200	135,478	0,511786	**-16,5**		
2.42a	-33,155	136,447	0,511854	**-15,1**		
2.42b	-33,032	136,103	0,511860	**-15,0**		
2,43	-33,032	136,602	0,511804	**-16,1**		
2,44	-31,789	141,186	0,512074	**-10,8**		
2,45	-32,051	142,371	0,511925	**-13,8**		
2.47	-32,355	142,401	0,512070	**-10,9**		
2,49	-32,507	143,012	0,512287	**-6,7**		
			**Average**	**-12,0**	**±**	**2,9**

Table S2. Neodymium isotopic composition of core MD03-2607 fine-grained detrital fractions

Depth (cm)	Age (kyrBP)	$^{143}Nd/^{144}Nd$	2 se	ε_{Nd}		
158	16,60	0,512389	0,000003	-4,85	±	0,10
210	19,06	0,512317	0,000005	-6,26	±	0,17
220	19,38	0,512302	0,000006	-6,55	±	0,17
230	19,71	0,512314	0,000007	-6,33	±	0,17
240	20,03	0,512317	0,000009	-6,26	±	0,17
250	20,37	0,512294	0,000008	-6,72	±	0,17
260	20,71	0,512307	0,000007	-6,46	±	0,17
270	21,17	0,512295	0,000007	-6,69	±	0,17
280	21,63	0,512286	0,000005	-6,87	±	0,17
290	21,94	0,512285	0,000010	-6,89	±	0,17
300	22,24	0,512283	0,000006	-6,92	±	0,17
310	22,72	0,512295	0,000005	-6,69	±	0,17
320	23,21	0,512273	0,000005	-7,12	±	0,17
330	23,69	0,512275	0,000004	-7,09	±	0,17
340	24,17	0,512289	0,000005	-6,80	±	0,17
350	24,66	0,512284	0,000006	-6,90	±	0,17
360	25,14	0,512269	0,000005	-7,19	±	0,17
370	26,68	0,512253	0,000005	-7,51	±	0,17
380	28,22	0,512244	0,000006	-7,68	±	0,17
390	29,76	0,512277	0,000005	-7,05	±	0,17
400	31,29	0,512298	0,000007	-6,64	±	0,17
410	32,83	0,512281	0,000005	-6,97	±	0,17
420	34,37	0,512292	0,000007	-6,74	±	0,17
430	35,91	0,512295	0,000005	-6,69	±	0,17
440	36,04	0,512307	0,000007	-6,45	±	0,17
449	36,15	0,512313	0,000007	-6,35	±	0,17
460	36,30	0,512309	0,000004	-6,42	±	0,10
468,5	36,41	0,512289	0,000006	-6,81	±	0,10
490	36,68	0,512314	0,000004	-6,33	±	0,10
498	37,39	0,512333	0,000003	-5,95	±	0,10
510	38,45	0,512321	0,000004	-6,18	±	0,10
519	39,25	0,512305	0,000004	-6,49	±	0,10
528	40,04	0,512308	0,000003	-6,44	±	0,10
540	40,88	0,512302	0,000004	-6,55	±	0,10
549	41,50	0,512292	0,000004	-6,76	±	0,10
557,5	42,09	0,512311	0,000003	-6,37	±	0,10
570	42,96	0,512287	0,000003	-6,84	±	0,10
579	43,59	0,512309	0,000004	-6,42	±	0,10
588	44,21	0,512322	0,000004	-6,17	±	0,10
599	44,97	0,512333	0,000003	-5,94	±	0,10
611	45,81	0,512337	0,000003	-5,86	±	0,10
620	46,43	0,512346	0,000004	-5,70	±	0,10

Table S2. (continued)

630	47,13	0,512323	0,000004	-6,15	±	0,10
640	47,82	0,512321	0,000005	-6,19	±	0,10
648,5	48,41	0,512324	0,000003	-6,13	±	0,10
660	49,21	0,512305	0,000004	-6,50	±	0,10
671	49,97	0,512314	0,000003	-6,33	±	0,10
680	50,60	0,512298	0,000004	-6,63	±	0,10
690	51,29	0,512289	0,000003	-6,81	±	0,10
701	52,06	0,512284	0,000005	-6,90	±	0,10
710	52,68	0,512295	0,000003	-6,70	±	0,10
720	53,38	0,512286	0,000004	-6,87	±	0,10
730	54,07	0,512292	0,000004	-6,75	±	0,10
740	54,76	0,512291	0,000003	-6,78	±	0,10
749	55,39	0,512284	0,000004	-6,91	±	0,10
762	56,29	0,512307	0,000003	-6,46	±	0,10
770	56,85	0,512348	0,000006	-5,66	±	0,17
780	57,54	0,512345	0,000006	-5,72	±	0,17
790	58,24	0,512353	0,000005	-5,56	±	0,17
800	58,93	0,512356	0,000005	-5,50	±	0,17
810	60,43	0,512353	0,000007	-5,57	±	0,17
820	61,93	0,512359	0,000005	-5,44	±	0,17
830	63,42	0,512366	0,000006	-5,31	±	0,17
840	64,92	0,512364	0,000007	-5,34	±	0,17
850	66,42	0,512361	0,000009	-5,40	±	0,17
860	67,92	0,512344	0,000008	-5,74	±	0,17
870	69,42	0,512311	0,000006	-6,38	±	0,17
880	70,91	0,512302	0,000004	-6,56	±	0,17
890	72,41	0,512286	0,000006	-6,87	±	0,17
900	73,91	0,512250	0,000007	-7,56	±	0,17
910	75,35	0,512252	0,000006	-7,53	±	0,17
920	76,79	0,512228	0,000006	-8,00	±	0,17
930	78,22	0,512229	0,000009	-7,98	±	0,17
940	79,66	0,512219	0,000005	-8,16	±	0,17
950	80,53	0,512214	0,000007	-8,27	±	0,17
960	81,40	0,512207	0,000005	-8,41	±	0,17
970	82,27	0,512189	0,000007	-8,76	±	0,17
980	83,14	0,512197	0,000007	-8,61	±	0,17
990	84,01	0,512183	0,000007	-8,88	±	0,17
1000	84,88	0,512151	0,000007	-9,50	±	0,17
1010	85,75	0,512136	0,000007	-9,80	±	0,17
1020	86,62	0,512175	0,000010	-9,03	±	0,17
1030	87,49	0,512028	0,000008	-11,91	±	0,17
1040	88,36	0,512233	0,000008	-7,90	±	0,17
1361	124,50	0,512129	0,000004	-9,92	±	0,10

References

Bouvier, A., Vervoort, J. D. & Patchett, P. J. The Lu–Hf and Sm–Nd isotopic composition of CHUR: constraints from unequilibrated chondrites and implications for the bulk composition of terrestrial planets. *Earth Planet. Sci. Lett.* **273**, 48–57 (2008).

Gingele, F. X., & De Deckker, P. Clay mineral, geochemical and Sr-Nd isotopic fingerprinting of sediments in the Murray-Darling fluvial system, southeast Australia. *Aust. J. Earth Sci.* **52**, 965-974 (2005).

Gingele, F. X, De Deckker, P., & Norman, M. Late Pleistocene and Holocene climate of SE Australia reconstructed from dust and river loads deposited offshore the River Murray mouth. *Earth Planet. Sci. Lett.* **255**, 257-272 (2007).

Lisiecki, L.E. and Raymo, M.E. A Pkuocene-Pleistocene stack of 57 globally distributed benthic $\delta^{18}O$ records. *Paleoceanography* **20**, 1-17. doi:10.1029/2004PA001071 (2005).

Lopes dos Santos, R. A. *et al.* Late Quaternary productivity changes from offshore Southeastern Australia: A biomarker approach. *Palaeogeogr. Palaeoclim. Palaeoecol.* **363/364**, 48-56 (2012).

Ménabréaz, L., Bourlès, D. L., Thouveny, N. Amplitude and timing of the Laschamp geomagnetic dipole low from the global atmospheric ^{10}Be overproduction: Contribution of authigenic $^{10}Be/^{9}Be$ ratios in west equatorial Pacific sediments. *J. Geophys. Res.* **117**, B11101, doi:10.1029/2012JB009256 (2012).

Tachikawa, K. *et al.* The precession phase of hydrological variability in the Western Pacific Warm Pool during the past 400 ka. *Quat. Sci. Rev.* **30**, 3716-3727 (2011).

Simon, Q. *et al.* Authigenic $^{10}Be/^{9}Be$ ratio signatures of the cosmogonic nuclide production linked to geomagnetic dipole moment variation since the Brunhes/Matuyama boundary. *J. Geophys. Res.*, n/a-n/a, doi:10.1002/2016JB013335 (2016).
